# Quorum quenching enzymes disrupt bacterial communication in a sex‐ and dose‐dependent manner

**DOI:** 10.1002/ame2.12520

**Published:** 2025-02-13

**Authors:** Aneesh Syal, Maria Martell, Rakesh Sikdar, Matthew Dietz, Zachary Ziegert, Cyrus Jahansouz, Mikael H. Elias, Christopher Staley

**Affiliations:** ^1^ Division of Basic and Translational Research, Department of Surgery University of Minnesota Minneapolis Minnesota USA; ^2^ BioTechnology Institute University of Minnesota St. Paul Minnesota USA; ^3^ Department of Biochemistry, Molecular Biology, and Biophysics University of Minnesota St. Paul Minnesota USA; ^4^ Division of Colon and Rectal Surgery, Department of Surgery University of Minnesota Minneapolis Minnesota USA

**Keywords:** microbiome, microbiota therapeutics, obesity, quorum sensing

## Abstract

**Background:**

Over the past 50 years, the incidence of obesity has gradually increased, necessitating investigation into the multifactorial contributors to this disease, including the gut microbiota. Bacteria within the human gut microbiome communicate using a density‐dependent process known as quorum sensing (QS), in which autoinducer (AI) molecules (e.g., *N‐*acyl‐homoserine lactones [AHLs]) are produced to enable bacterial interactions and regulate gene expression.

**Methods:**

We aimed to disrupt QS using quorum quenching (QQ) lactonases GcL and SsoPox, which cleave AHL signaling molecules in a taxa‐specific manner based on differing enzyme affinities for different substrates. We hypothesized that QQ hinders signals from obesity‐associated pathobionts, thereby slowing or preventing obesity.

**Results:**

In a murine model of diet‐induced obesity, we observed GcL and SsoPox treatments have separate sex‐dependent and dose‐dependent effects on intestinal community composition and diversity. Notably, male mice given 2 mg/mL SsoPox exhibited significant changes in the relative abundances of gram‐negative taxa, including Porphyromonadaceae, Akkermansiaceae, Muribaculaceae, and Bacteroidales (Kruskal–Wallis *p* < 0.001). Additionally, we used covariance matrix network analysis to model bacterial taxa co‐occurrence due to QQ enzyme administration. There were more associations among taxa in control mice, particularly among gram‐negative bacteria, whereas mice receiving SsoPox had the fewest associations.

**Conclusions:**

Overall, our study establishes proof of concept that QQ is a targetable strategy for microbial control in vivo. Further characterization and dosage optimization of QQ enzymes are necessary to harness their therapeutic capability for the treatment of chronic microbial‐associated diseases.

## Graphical abstract

Male and female mice were treated with the quorum quenching (QQ) enzymes SsoPox and GcL in drinking water at concentrations of 1 and 2 mg/mL (or control) for 6 weeks while on a Western diet. Mice received food and water ad libitum, and mouse weights were measured to determine if QQ could block diet‐induced obesity. The fecal microbiome was characterized by Illumina amplicon sequencing of the 16S ribosomal RNA gene to evaluate changes in microbial composition resulting from QQ.

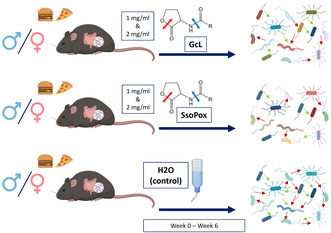

## 1 INTRODUCTION

Over the past 50 years, the incidence of obesity has gradually increased, and since 1980, the prevalence of obesity has more than doubled, leaving nearly 700 million people affected by the disease worldwide.^1^, ^2^, ^3^, ^4^ Obesity adversely impacts numerous physiological functions while predisposing patients to several comorbidities, including atherosclerosis,^2^ diabetes,^3^ and certain cancers.^1^, ^2^, ^5^ If these trends persist, roughly 20% of the global adult population will be obese by 2030, placing significant economic and social burdens on health care systems.^6^ Although the primary cause of obesity is the accumulation of excess body fat, recent findings have identified obesity as a complex, multifactorial disease, linking the metabolism with behavioral, genetic, environmental, and socioeconomic factors.^1^, ^7^, ^8^, ^9^, ^10^

The intestinal microbiota is at the center of the multifactorial interactions driving obesity and plays a role in regulating host weight and metabolism.^10^, ^11^ Previous studies comparing conventionalized germ‐free mice to nonconventionalized, germ‐free mice indicated a difference between the intestinal microbiota from obese and lean mice.^12^, ^13^ One study by Ridaura et al.,^14^ in which the microbiota from twins discordant for obesity was transplanted into germ‐free mice, found the lean microbiota was more invasive compared to the obese microbiota.^14^ Specifically, when mice with lean and obese phenotypes were cohoused, microbiota associated with lean phenotypes (e.g., members of the Bacteroidetes) were able to displace obese‐associated microbiota, but lean mice resisted incorporation of obese microbiota,^14^ suggesting that beneficial microbial populations may outcompete those associated with obesity. Recent studies have also shown that dysbiosis, an imbalance of microbiota composition and function, may increase the risk of developing chronic diseases, including inflammatory bowel disease, cancer, and obesity.^15^, ^16^, ^17^ Moreover, obese microbiomes are extremely resilient, even after dietary and lifestyle interventions.^18^ These findings indicate that the intestinal microbiota plays both an associative and causative role in driving and maintaining obesity while highlighting the need to understand the mechanisms by which the microbiota influences the disease.

Bacteria communicate through diffusible, small molecules that regulate gene expression in a density‐dependent manner and based on surrounding environmental conditions, called quorum sensing (QS).^19^ The accumulation of autoinducer (AI) molecules, such as *N‐*acyl‐homoserine lactones (AHLs; AI‐1), by gram‐negative bacteria is a sensitive and effective way to regulate gene expression and plays a significant role in intra‐ and interspecies, as well as inter‐kingdom, interactions.^20^ In addition to AHLs, produced and/or sensed predominantly by gram‐negative Proteobacteria and some Bacteroidetes, Cyanobacteria, and Archaea,^21^, ^22^, ^23^ gram‐positive bacteria produce and respond to oligopeptide AIs. Additionally, AI‐2, a furanosyl borate diester, a universal signal of interspecies communication, is produced and/or sensed by both gram‐negative and gram‐positive bacteria. Moreover, discovery and characterization of new classes of QS compounds remain an active area of research.^24^, ^25^, ^26^ Although some signals, like AI‐2, may be used for “signaling,” or the induction of an evolved behavior in the presence of QS molecules, they may also be “cues,” or used to manipulate or coerce specific behaviors from other species.^27^ Importantly, the host also monitors bacterial QS via the aryl hydrocarbon receptor^28^ and, in response, regulates host defenses, including obesity‐associated inflammation, potentially mediating an obese phenotype.^29^

In this study, we hypothesized that disrupting QS signaling, a process termed quorum quenching (QQ),^30^, ^31^ may be a targetable strategy to turn off signaling from obesity‐associated pathobionts to prevent or slow obesity progression. The AHL signaling molecules produced are specific to individual taxa and vary in length of the carbon chain^32^; therefore, selecting lactonases with differing specificity is likely to have different effects throughout the microbial community.^33^ We tested two lactonases: SsoPox, a thermostable lactonase, which acts on longer‐chain AHLs (C8–C12),^34^ and GcL, a highly proficient, promiscuous lactonase with broad substrate preference for AHLs (C4–C12).^32^, ^33^ These lactonases were delivered in drinking water, and mice were fed a Western diet (WD) for 6 weeks to determine the effects of QQ enzymes on the microbial communities, food intake, and weight gain. Results from this study provide one of the first demonstrations of the use of QQ enzymes in vivo and suggest potential future therapeutic applications of these molecules.

## 2 METHODS

### 2.1 Production of QQ lactonases SsoPox and GcL

The mutant lactonase SsoPox W263I,^35^ referred to as SsoPox throughout this manuscript, and wild‐type GcL with an *N*‐terminal Strep‐tag II^36^ were overexpressed in *Escherichia coli* strain BL21 Star (DE3) (Invitrogen, Carlsbad, CA, USA) containing the pGro7 plasmid (TakaRa Bio, San Jose, CA, USA). Enzymes were produced using previously described procedures^37^, ^38^ adapted to a 75‐L fermentation system (New Brunswick Scientific, Edison, NJ, USA). Briefly, exponential phase growing cultures in fermentation medium at 30°C (with 300 rpm agitation, 5 psi vessel pressure, 25 slpm air flow, dissolved oxygen >30%, and pH 6.8 with continuous glycerol feed) were induced at 23°C with 0.2% arabinose, 0.2 mM CoCl_2_, and 0.25 mM IPTG (Isopropyl ß‐D‐1‐thiogalactopyranoside) and harvested after 25 h. Cells were lysed in 20 mM Tris‐Cl pH 8.0 buffer containing 2 mM MgCl_2_ and 11 KU of Benzonase nuclease using Panther homogenizer (15 000 psi primary, 1500 psi secondary; GEA Process Engineering, Inc., Hudson, WI, USA). Lysed cells were centrifuged, and clarified supernatants were subjected to heat treatment at 75°C (for SsoPox W263I) or 65°C (for GcL) for 30 min and centrifuged (15 000 g/30 min/4°C) to remove precipitants. The clarified protein solution was ultrafiltered (0.6 μm for GcL; 0.6 μm followed by 0.2‐μm nominal filter for SsoPox) concentrated and diafiltered (using 5‐kDa MWCO diafiltration membrane) in 50‐mM Tris‐Cl, pH 8.5 buffer and subsequently lyophilized under vacuum using a VirTis FreezeMobile 25 system (ATS Scientific Products, Warminster, PA, USA). The lyophilized enzymes were reconstituted in deionized water at indicated concentrations and filter‐sterilized immediately before use. Both enzymes were assayed for lactonase activity against 5‐thiobutyl‐γ‐butyrolactone (TBBL) substrate (synthesized by Enamine LTD, Kyiv, Ukraine) in an activity buffer (50‐mM HEPES pH 8.0, 150‐mM NaCl, 0.2‐mM CoCl_2_) containing 0.5‐mM TBBL and 1‐mM Ellman's reagent (5,5′‐dithiobis‐[2‐nitrobenzoic acid] or DTNB).^39^

### 2.2 Mice and QQ intervention

Male and female specific‐pathogen‐free (SPF) C57BL/6 mice (*n* = 54) purchased from Jackson Laboratories (Bar Harbor, ME, USA) at 6 weeks of age were conventionally housed, individually, in AAALAC‐approved animal facilities at the University of Minnesota. Mice were randomly assigned to a 6‐week treatment period of either drinking water (*n* = 18), GcL (*n* = 18), or SsoPox (*n* = 18). Three mice in each treatment group received 2 mg/mL QQ enzymes; all others received 1 mg/mL enzyme or unamended drinking water (Table 1). Mice were continuously fed Western chow (Teklad diet 88 137%—15.2%, 42.7%, and 42.0% kcal from protein, carbohydrates, and fat, respectively), similar in composition to that of humans,^40^ and water was also available ad libitum throughout the 6‐week study period. Fecal sample collections and weight and food intake monitoring were performed weekly. After the mice were transferred to new cages, 2 h was given before fresh fecal samples were collected. Mice were housed under a 12‐h light–dark cycle at 23°C. Experimental procedures were approved by the University of Minnesota Institutional Animal Care and Use Committee (IACUC) and performed following the Office of Laboratory Animal Welfare guidelines.

### 2.3 DNA extraction and sequencing

Intestinal microbiota was characterized from fecal pellets collected at six time points, corresponding to each week of the experiment. Bacterial DNA was extracted from individual mouse fecal pellets (approximately 0.1 g) using the DNeasy PowerSoil Pro DNA Isolation Kit (QIAGEN, Hilden, Germany) on the automated QIAcube platform (QIAGEN) using the inhibitor removal technology (IRT) protocol. Using the 515F/806R primer set,^41^ the V4 hypervariable region of the 16S ribosomal RNA (rRNA) gene was amplified and paired‐end sequenced on the MiSeq platform (Illumina, Inc., San Diego, CA, USA) at a read length of 301 nucleotides (nt) by the University of Minnesota Genomics Center (UMGC, Minneapolis, MN, USA).^42^, ^43^ Raw sequencing data were deposited in the Sequence Read Archive under BioProject accession number SRP415730.^44^

### 2.4 16S rRNA amplicon processing and analysis

All sequencing data were processed and analyzed using mothur (version 1.41.1)^45^ and a modified version of our previously published processing pipeline,^46^ with an initial read cut to 170 nt. Sequences were pair‐end‐joined and trimmed for quality. Clustering was performed by aligning sequences against the SILVA database (version 138.1).^47^ A 2% pre‐cluster was used to remove sequences likely to contain errors.^48^ Chimeras were identified and removed using UCHIME (version 4.2.40).^49^ Amplicon sequence variants (ASVs) were binned at 99% sequence similarity using OptiClust clustering,^50^ and classified against the version 18 release from the Ribosomal Database Project.^51^ All samples from experiments involving the administration of 1 mg/mL of QQ enzyme were processed through MMUPHin (Meta‐analysis Methods with Uniform Pipeline for Heterogeneity in Microbiome Studies).^52^ MMUPHin allowed for the normalization and combination of replicated 1 mg/mL experiments to help reduce batch effects. Alpha diversity (within‐sample diversity) and beta diversity (between‐sample diversity) were calculated as the Shannon and Chao1 indices and Bray–Curtis dissimilarities,^53^ respectively, using mothur.

### 2.5 Statistical analysis

For statistical comparisons, samples were rarefied to 4800 reads per sample by random subsampling. Among all samples, a mean estimated Good's coverage of 98.87 ± 0.01% was achieved following rarefaction. Data are presented as means ± standard deviation. Differences in alpha diversity were evaluated using ANOVA with Tukey's HSD procedure for pairwise comparisons. Differences in the relative abundances of families were determined using the Kruskal–Wallis nonparametric test using the Steel‐Dwass‐Critchlow‐Fligner procedure for pairwise comparisons. ANOVA and Kruskal–Wallis tests were completed using XLSTAT software (version 2022.5.1; Lumivero, Denver, CO, USA). Overall community comparisons were evaluated by ANOSIM,^54^ and samples were visualized by ordination using PCoA of Bray–Curtis distances.^55^ Correlation of family abundances with axes positions was performed using Spearman correlation tests. ANOSIM, PCoA, and Spearman correlation tests were performed using mothur. All statistics were evaluated at *α* = 0.05, with Bonferroni corrections for multiple comparisons. To create covariance matrix estimation networks, we used the R package Sparse and Positive Definite Covariance Matrix Estimation with Compositional Data (SpPDCC) [https://github.com/ajmolstad/SpPDCC].^56^ This package allowed us to estimate basis covariance matrices from our compositional data through a proximal–proximal descent algorithm. For the network involving samples from mice receiving 1 mg/mL QQ enzyme, we used only the week 6 samples to allow for endpoint comparisons.

## 3 RESULTS

### 3.1 QQ enzymes do not significantly impact weight gain or food intake in Western diet–fed mice

Administration of the WD in mice led to significant weight gain among all treatment groups (Table 1). Control (DW) mice gained an average of 7.65 ± 1.49 g during the 6‐week study period. Similarly, mice receiving 1 mg/mL of GcL enzyme gained an average of 13.09 ± 0.55 g, whereas mice administered 2 mg/mL of GcL enzyme gained an average of 6.62 ± 3.44 g by week 6. Further, mice administered 1 mg/mL of SsoPox gained an average of 6.76 ± 1.65 g, and mice administered 2 mg/mL of SsoPox gained an average of 9.95 ± 3.90 g by the end of the experiment (Table 1). The average weight of all mice changed significantly from the baseline (week 0) to week 6 when the weight data from both the 1‐ and 2 mg/mL QQ enzyme experiments were combined (analysis of variance [ANOVA] *F* = 179.63, *p* < 0.001; Table 1); however, no pairwise differences were observed among treatment groups (Tukey's post‐hoc *p* > 0.05). Furthermore, no differences were observed in food intake between treatment groups and time points when combining the 1‐ and 2 mg/mL QQ enzyme experiments (ANOVA *F* = 0.435, *p* = 0.660; Table 1).

**TABLE 1:** Summary of physiological variables.

Treatment	Dose	Sex	*n*	Weight (g)			Food intake (g)		
				Week 0	Week 6	Δ	Week 0	Week 6	Δ
DW	N/A	Male	9	22.81 ± 1.17	34.33 ± 3.53	11.52 ± 2.35	31.88 ± 7.46	26.97 ± 5.36^B^	−4.92 ± 2.10
	N/A	Female	9	18.43 ± 1.01	22.22 ± 1.64	3.79 ± 0.63	27.63 ± 3.65	47.17 ± 23.21	19.53 ± 19.55
GcL	1 mg/mL	Male	6	23.22 ± 1.10	33.88 ± 2.14	10.67 ± 1.04	30.80 ± 5.13	29.30 ± 7.48^AB^	−1.50 ± 2.35
	1 mg/mL	Female	6	17.30 ± 1.16	22.13 ± 1.11	4.83 ± 0.05	29.97 ± 14.84	41.70 ± 14.00	11.73 ± 0.84
	2 mg/mL	Male	3	24.10 ± 1.28	33.33 ± 4.18	9.23 ± 2.90	36.27 ± 9.35	34.30 ± 10.11^AB^	−1.97 ± 0.76
	2 mg/mL	Female	3	17.77 ± 0.55	21.77 ± 2.35	4.00 ± 1.80	24.93 ± 1.01	46.80 ± 15.24	21.87 ± 14.22
SsoPox	1 mg/mL	Male	6	23.20 ± 1.06	32.68 ± 3.24	9.48 ± 2.18	34.00 ± 1.49	27.95 ± 2.05^AB^	−6.05 ± 0.56
	1 mg/mL	Female	6	17.50 ± 0.50	21.53 ± 1.60	4.03 ± 1.11	26.70 ± 1.13	54.80 ± 26.07	28.10 ± 24.94
	2 mg/mL	Male	3	23.43 ± 0.42	34.87 ± 1.90	11.43 ± 1.50	28.60 ± 2.44	48.43 ± 13.50^A^	19.83 ± 11.05
	2 mg/mL	Female	3	18.50 ± 1.48	26.97 ± 8.00	8.47 ± 6.52	26.17 ± 3.47	44.93 ± 26.31	18.77 ± 22.84
*p*‐value		Male		0.521	0.836	‐	0.636	0.034	‐
		Female		0.170	0.115	‐	0.896	0.972	‐

*Note*: Values reflect mean ± standard deviation. Mean weight, change in weight, food intake, and change in food intake of mice receiving drinking water, GcL, and SsoPox between weeks 0 and 6 are shown and separated by quorum quenching (QQ) dosage (1 mg/mL vs. 2 mg/mL). Δ indicates change from week 0 to week 6. All statistical analyses were performed using an analysis of variance (ANOVA) test evaluated at *α* = 0.05. Samples sharing the same letter (^AB^) did not differ significantly in pairwise comparisons using Tukey's post‐hoc test (*p* > 0.05).

### 3.2 QQ treatment alters community composition

Because the baseline sample (week 0) was collected prior to the initiation of WD, it was excluded from temporal analyses to better capture the effects of the individual QQ enzymes in relation to diet‐induced obesity. After baseline samples were excluded, no significant temporal variation was observed between weeks 1 and 6 among all treatment groups receiving 1‐ or 2 mg/mL QQ enzyme (analysis of similarities [ANOSIM] *R* = 0.005 and 0.002, *p* = 0.29 and 0.394, respectively). Therefore, temporal variation was not considered in further analyses, and all samples, excluding baseline, were combined to increase statistical power.

#### 3.2.1 Lower QQ enzyme dose (1 mg/mL)

**FIGURE 1:**
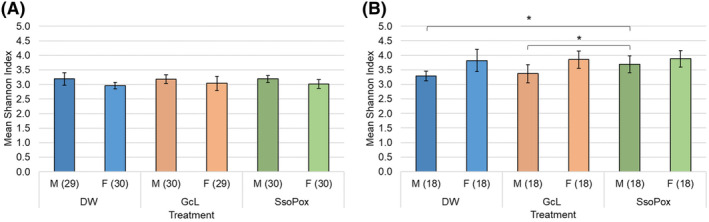
Mean Shannon index of bacterial taxa in fecal samples. Mean alpha diversities according to the Shannon index across various treatment groups, sex, and quorum quenching (QQ) enzyme dosages are shown. (A) 1 mg/mL and (B) 2 mg/mL are shown. Error bars indicate the mean ± standard deviation. Significant differences in Shannon index, as determined by ANOVA, are indicated by asterisk (*, Tukey's post‐hoc *p* < 0.05).

**FIGURE 2:**
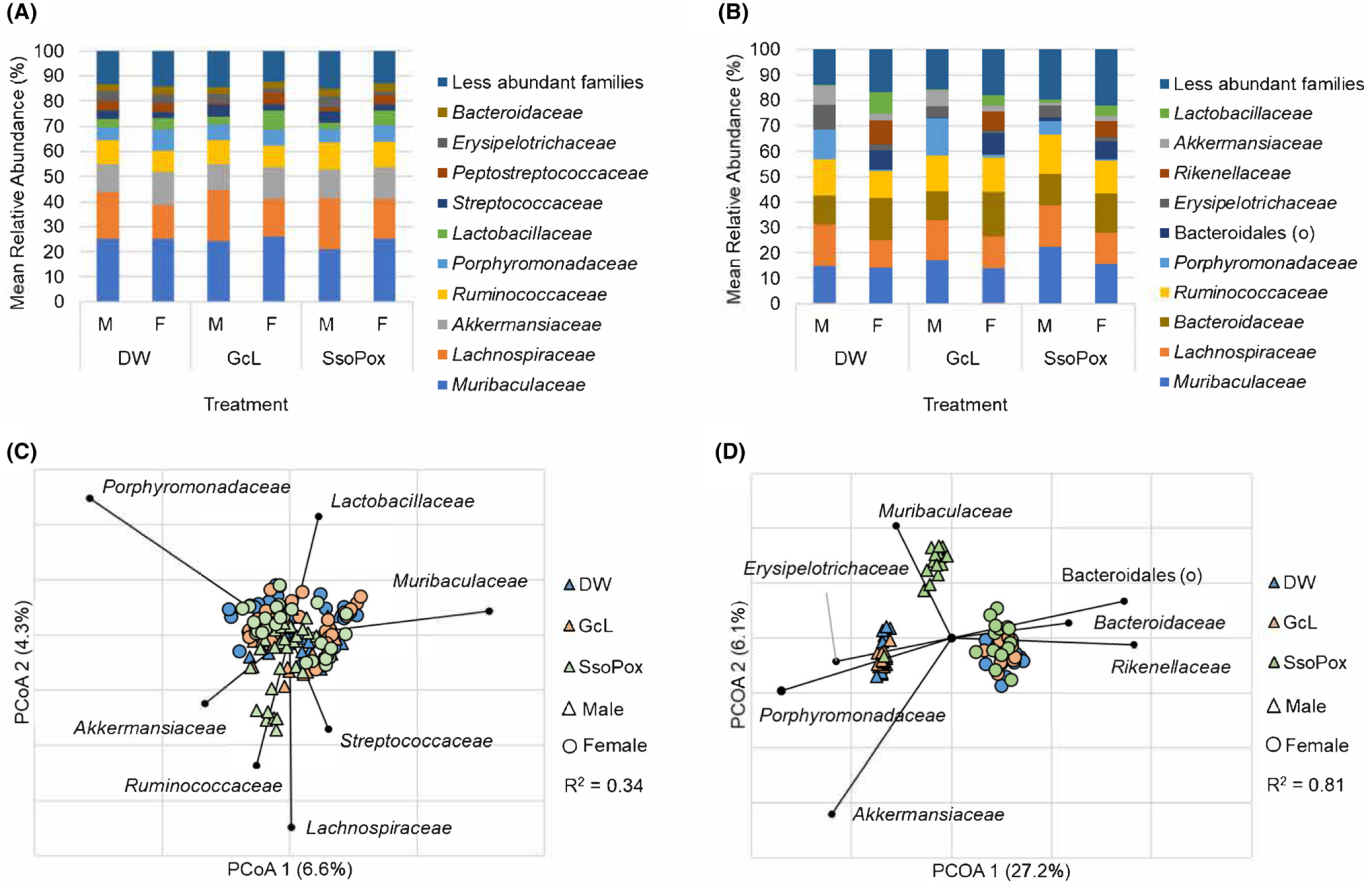
Bacterial communities in males and females. (A, B) Predominant families for each experimental treatment and sex, for mice receiving 1‐ and 2 mg/mL quorum quenching (QQ) enzyme, respectively. Less‐abundant genera comprised <15.1% and <22.1% of total taxonomic composition, respectively. (C) Principal coordinate analysis (PCoA) of Bray–Curtis dissimilarities among samples from mice receiving 1 mg/mL QQ enzyme. Families significantly correlated to axis position by Spearman correlation (*p* < 0.05) are plotted on the PCoA with vector length indicating magnitude of correlation. (D) PCoA of Bray–Curtis dissimilarities among samples from mice receiving 2 mg/mL QQ enzyme. Families significantly correlated to axis position by Spearman correlation are plotted on the PCoA, with vector length indicating magnitude of correlation.

There were no significant differences in Shannon alpha diversity when comparing mice receiving 1 mg/mL of QQ enzyme by treatment group and sex (ANOVA *F =* 2.01, *p =* 0.140; Figure 1A). Similarly, no significant differences were observed when Chao1 indices were compared by treatment group and sex (ANOVA *F* = 0.12, *p =* 0.883). Male mice receiving GcL had significantly lower relative abundances of Peptostreptococcaceae and Erysipelotrichaceae than those observed in control mice (Steel‐Dwass‐Critchlow‐Fligner post‐hoc *p* < 0.05 and <0.0001; Figure 2A; Table 2). Similarly, in females receiving GcL, the relative abundances of Erysipelotrichaceae were significantly lower than in control mice (post‐hoc *p* < 0.0001; Figure 2A; Table 2). Male mice receiving SsoPox did not display significant changes in the relative abundances of predominant families relative to drinking water mice. Similar to females receiving GcL, female mice receiving SsoPox had significantly lower relative abundances of Erysipelotrichaceae compared to controls (post‐hoc *p* < 0.0001; Figure 2A; Table 2).

**TABLE 2:** Abundances of predominant families that varied significantly among mice receiving 1 mg/mL quorum quenching (QQ) enzyme or drinking water by Kruskal–Wallis test.

Family	DW		GcL		SsoPox		*p*‐value
	Male	Female	Male	Female	Male	Female	
Muribaculaceae	25.31 ± 3.86	25.24 ± 6.80	24.33 ± 5.27	25.96 ± 6.42	21.08 ± 8.80	25.03 ± 7.69	0.59
Lachnospiraceae	18.57 ± 4.47^BCD^	13.30 ± 3.92^A^	20.35 ± 6.81^CD^	15.16 ± 4.85^AB^	20.32 ± 3.89^D^	15.95 ± 4.60^ABC^	0.0001
Akkermansiaceae	11.10 ± 4.93	13.33 ± 5.98	10.13 ± 5.34	12.42 ± 5.87	11.48 ± 5.05	12.63 ± 5.38	0.35
Ruminococcaceae	9.62 ± 2.67	8.27 ± 2.41	9.62 ± 3.31	8.71 ± 2.50	10.63 ± 4.06	10.35 ± 4.30	0.06
Porphyromonadaceae	5.08 ± 4.13	8.65 ± 7.08	6.41 ± 5.35	6.38 ± 5.37	5.54 ± 4.17	6.46 ± 5.54	0.09
Lactobacillaceae	3.20 ± 4.86	4.53 ± 3.42	3.10 ± 3.03	7.60 ± 4.41	2.38 ± 1.66	5.88 ± 7.18	0.09
Streptococcaceae	3.46 ± 1.66^B^	2.12 ± 0.72^A^	4.56 ± 2.10^B^	2.38 ± 0.88^A^	4.47 ± 2.02^B^	2.36 ± 1.12^A^	0.0001
Peptostreptococcaceae	3.85 ± 4.09^B^	3.78 ± 4.05^AB^	0.75 ± 1.90^A^	4.70 ± 4.66^B^	1.5 ± 3.10^AB^	3.8 ± 3.56^B^	0.0001
Erysipelotrichaceae	3.92 ± 2.24^C^	3.23 ± 1.90^C^	3.47 ± 3.01^BC^	1.7 ± 1.10^AB^	4.49 ± 3.00^C^	1.26 ± 0.58^A^	0.003
Bacteroidaceae	2.59 ± 1.41	3.48 ± 2.56	2.65 ± 1.82	2.64 ± 1.98	3.07 ± 1.63	3.14 ± 2.58	0.353

*Note*: Values reflect mean ± standard deviation. Pairwise comparisons were calculated using the Steel‐Dwass‐Critchlow‐Fligner procedure. Samples sharing the same letter (^ABCD^) did not differ significantly in pairwise comparisons using the Steel‐Dwass‐Critchlow‐Fligner procedure (*p* > 0.05).

Overall community composition (beta diversity), measured using Bray–Curtis dissimilarities, differed significantly between male and female mice in each of the control, GcL, and SsoPox groups (ANOSIM *R* = 0.18, 0.21, and 0.16, respectively, *p* < 0.001; Figure 2C). However, among mice of the same sex between treatment groups, differences in beta diversity remained statistically insignificant (pairwise *R* = 0.05–0.15, *p* > 0.003, Bonferroni‐corrected *α* = 0.003; Figure 2C). Principal coordinate analysis (PCoA) of male and female microbial communities revealed their axis positions were significantly correlated with the relative abundances of Porphyromonadaceae, Lactobacillaceae, Muribaculaceae, Akkermansiaceae, Ruminococcaceae, Lachnospiraceae, and Streptococcaceae (Spearman *p* < 0.05; Figure 2C).

**FIGURE 3:**
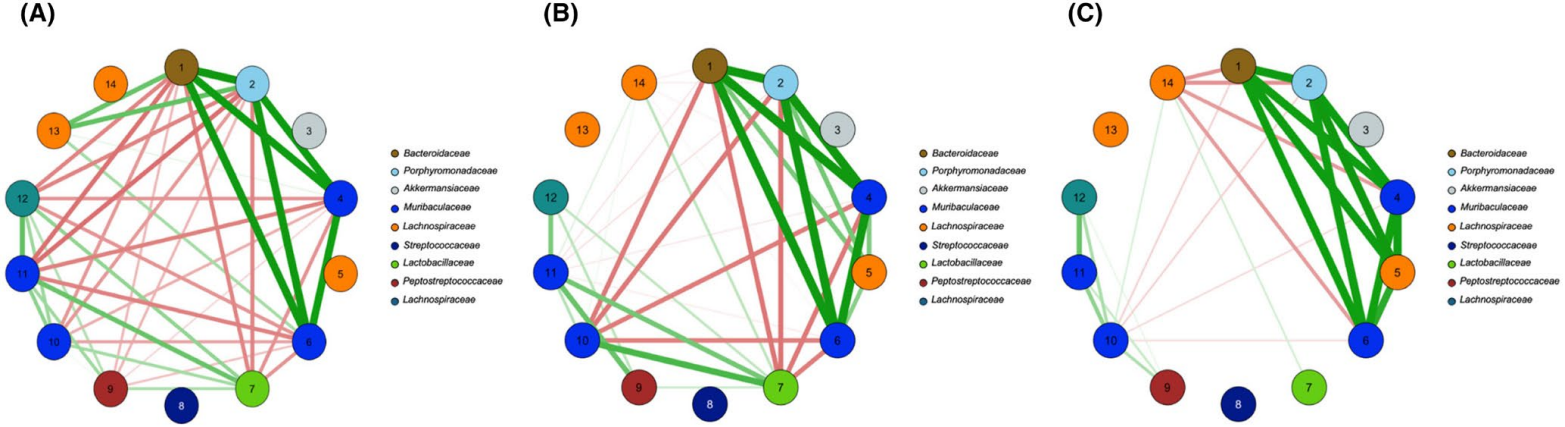
Estimated covariance matrices at study endpoint of samples from mice receiving 1 mg/mL QQ enzyme. (A) Control mice. (B) GcL mice. (C) SsoPox mice. The thickness of the edge corresponds to the strength of the association where stronger associations are shown by thicker edges. Positive and negative correlations are colored green and red, respectively, and a zero correlation is indicated by the lack of an edge.

To gain more perspective on microbiome interactions occurring from the use of GcL or SsoPox, we applied a systems‐oriented approach by estimating covariance matrices for the (latent) log‐abundances of the microbes in each of our sample populations. To achieve a sufficient sample size for analysis, male and female samples were combined in each treatment group. Visual examination of the estimated covariance matrices revealed the control network and both treatment networks have differing strengths and number of associations between nodes (Figure 3A–C). Consistent, strong positive associations among Bacteroidaceae, Porphyromonadaceae, and Muribaculaceae were observed in all three networks. Further, between networks the number of associations was greatest in the control group (Figure 3A), whereas there were least associations in the SsoPox treatment group (Figure 3C). Thus, our covariance matrix models were consistent with our hypothesis—as QQ enzymes are administered, reduced communication within the microbiome appears to diminish patterns of co‐occurrence.

#### 3.2.2 Higher QQ enzyme dose (2 mg/mL)

To determine whether a higher dose may induce different changes in the intestinal microbiome, mice received 2 mg/mL of QQ enzyme. After the administration of 2 mg/mL QQ enzyme, Shannon index was significantly altered based on the treatment group among male mice, but not female mice (ANOVA *F* = 11.57 and 0.121, *p* < 0.0001 and 0.887, respectively; Figure 1B). Specifically, male mice receiving SsoPox had greater Shannon alpha diversities than male mice receiving either GcL or drinking water (Tukey's post‐hoc *p* < 0.05). However, no significant differences were observed in either sex when Chao1 indices were compared by treatment group (ANOVA *F* = 0.253–0.492, *p* = 0.615–0.778). Male mice receiving GcL had significantly higher relative abundances of Erysipelotrichaceae than in the control (Steel‐Dwass‐Critchlow‐Fligner post‐hoc *p* = 0.006; Figure 2B; Table 3). Female mice receiving GcL had no significant changes in the relative abundances of predominant families relative to drinking water mice. Male mice receiving SsoPox had significantly greater relative abundances of Muribaculaceae and Bacteroidales than those observed in the control, and significantly lower relative abundances of Porphyromonadaceae, Erysipelotrichaceae, and Akkermansiaceae compared to the controls (Steel‐Dwass‐Critchlow‐Fligner post‐hoc all *p* < 0.0001, Figure 2B; Table 3).

**TABLE 3:** Abundances of predominant families that varied significantly among mice receiving 2 mg/mL QQ enzyme or drinking water by Kruskal–Wallis test.

Treatment	DW		GcL		SsoPox		*p*‐value
Sex	Male	Female	Male	Female	Male	Female	
Muribaculaceae	14.66 ± 2.93^A^	14.28 ± 3.50 ^A^	17.09 ± 2.92^A^	13.84 ± 2.48^A^	22.3 ± 3.58^B^	15.77 ± 3.90^A^	0.0001
Lachnospiraceae	16.63 ± 3.08^C^	10.65 ± 2.98^A^	15.78 ± 3.83^BC^	12.57 ± 4.07^ABC^	16.34 ± 4.95^BC^	12.18 ± 3.78^AB^	0.0001
Akkermansiaceae	7.49 ± 3.69^B^	2.45 ± 3.58^A^	6.42 ± 3.95^B^	2.47 ± 4.19^A^	1.20 ± 2.94^A^	1.80 ± 2.42^A^	0.0001
Ruminococcaceae	14.40 ± 3.95	10.43 ± 4.96	14.05 ± 3.13	13.45 ± 4.93	15.39 ± 4.33	12.89 ± 3.79	0.016
Porphyromonadaceae	11.80 ± 2.07^C^	0.55 ± 0.95^A^	14.63 ± 4.14^C^	1.22 ± 1.38^A^	5.22 ± 3.39^B^	0.45 ± 0.36^A^	0.0001
Lactobacillaceae	0.48 ± 0.33^A^	8.73 ± 8.92^C^	0.47 ± 0.28^A^	3.95 ± 3.69^BC^	1.22 ± 1.11^AB^	4.29 ± 4.57^BC^	0.0001
Bacteroidales (o)	0.02 ± 0.01^A^	7.84 ± 1.36^C^	0.03 ± 0.04^A^	8.50 ± 1.90^C^	1.69 ± 0.91^B^	7.08 ± 2.14^C^	0.0001
Rikenellaceae	0.01 ± 0.01	9.31 ± 4.16^B^	0.0 ± 0.01^A^	7.27 ± 3.07^B^	0.02 ± 0.05^A^	6.32 ± 3.04^B^	0.0001
Erysipelotrichaceae	9.57 ± 3.80^C^	2.34 ± 2.80^AB^	4.42 ± 2.58^B^	1.19 ± 1.18^A^	4.44 ± 4.47^AB^	1.72 ± 1.9^A^	0.0001
Bacteroidaceae	11.15 ± 2.27^A^	16.74 ± 3.94^BC^	11.53 ± 2.41^A^	17.49 ± 4.62^C^	12.38 ± 3.23^AB^	15.49 ± 3.5^BC^	0.0001

*Note*: Values reflect mean ± standard deviation. Pairwise comparisons were calculated using the Steel‐Dwass‐Critchlow‐Fligner procedure. Samples sharing the same letter (^ABC^) did not differ significantly in pairwise comparisons using the Steel‐Dwass‐Critchlow‐Fligner procedure (*p* > 0.05).

In contrast to mice receiving 1 mg/mL of QQ enzyme, PCoA analysis revealed overall fecal community composition and diversity among mice receiving 2 mg/mL QQ enzyme differed significantly based on treatment group and sex (ANOSIM *R* = 0.74, *p* < 0.001; Figure 2D). Pairwise comparisons showed significant differences between male and female mice in each of the control, GcL, and SsoPox groups (pairwise *R* = 0.91–0.99, *p <* 0.001, Bonferroni‐corrected *α =* 0.003; Figure 2D). Male mice receiving GcL had significantly different community composition than controls (pairwise *R* = 0.28, *p* < 0.001, Bonferroni‐corrected *α* = 0.003; Figure 2D). Likewise, males receiving SsoPox had communities that differed significantly from male control mice (pairwise *R* = 0.76, *p <* 0.001; Figure 2D). Female mice receiving SsoPox, but not GcL, had communities that were significantly different compared to female controls (pairwise *R* = 0.24, *p* < 0.001; Figure 2D). PCoA of male and female microbial communities revealed their axis positions were significantly correlated with the relative abundances of Akkermansiaceae, Porphyromonadaceae, Erysipelotrichaceae, Muribaculaceae, Bacteroidales, Bacteroidaceae, and Rikenellaceae (Spearman *p* < 0.05; Figure 2D). Covariance matrix analysis was not performed for 2 mg/mL experimental groups due to insufficient sample size for analysis.

## 4 DISCUSSION

Rising obesity rates and obesity‐associated metabolic disorders underscore the importance of exploring various contributors to this complex disease, including the intestinal microbiota.^1^, ^2^, ^3^, ^4^, ^5^ Given the prevalence of QS in the human gut microbiome,^57^ and the reported effect of QQ strategies on microbiome population structures,^26^ we aimed to use GcL and SsoPox, two QQ lactonases that degrade AHL signaling molecules from predominantly gram‐negative bacteria, to test the hypothesis that QQ hinders signals from obesity‐associated pathobionts and consequently slows or prevents the development of obesity.

In a murine model of diet‐induced obesity, GcL and SsoPox treatments produced separate, sex‐specific and dose‐dependent effects on intestinal community composition and diversity. Different bacterial taxa produce unique AHLs with differing lengths and compositions of acyl side chains, depending on the type(s) of synthase genes present.^32^, ^33^ Thus, the enzymes likely have differing effects on taxonomic composition due to GcL having a broader affinity for C4–C12 AHLs, whereas SsoPox has affinity for only longer‐chain C8–C12 AHLs.^26^ Using both enzymes, we observed alterations in the relative abundances of specific taxa such as Erysipelotrichaceae, Peptostreptococcaceae, Porphyromonadaceae, Akkermansiaceae, Muribaculaceae, and Bacteroidales without observable phenotypic effects, such as severe weight loss or stool changes. These results suggest that QQ lactonases are able to be administered in vivo in drinking water and have effects on the intestinal microbiota, supporting the premise that QQ lactonases may be able to influence host phenotype through AHL‐mediated modification of the microbiota. However, weight is a poor measure of metabolic dysregulation, and future studies assessing the effects of QQ on diet‐induced obesity should consider more sophisticated measures of body composition and metabolic activity.^58^

Previous research has found that AHL signaling molecules vary in length and in the composition of their acyl side chains, likely affecting the taxonomic specificity of this signal.^59^ QQ lactonases, SsoPox and GcL, act on long‐chain (C8–C12) or broad‐range (C4–C12) AHLs, respectively, suggesting that QQ enzymes may have the potential to target specific taxa by hydrolyzing the side chains of their signaling molecules, thereby disrupting their interactions with other bacteria.^35^, ^36^, ^59^ Our findings build on this prior knowledge by demonstrating that SsoPox and GcL have differential effects on the relative abundances of specific gram‐negative and gram‐positive bacteria, with these effects being potentially amplified with increasing dose. Collectively, these results indicate that QQ lactonases may have potential as a targetable approach to reduce the relative abundances of bacteria associated with obesity, without significantly disrupting the composition and diversity of the microbial ecosystem within the gut.

Further demonstrating the distinct effects of GcL and SsoPox, our covariance network analysis of taxa co‐occurrence revealed more associations among taxa in control mice, whereas mice receiving SsoPox had the fewest associations. Our analysis suggests GcL and SsoPox may also have differential effects on bacterial community networks, in which GcL may have an intermediate effect between SsoPox and the control.

Prior research suggests a bidirectional form of communication between the endocrine system and the gut microbiota.^60^ Regardless of dosage, our findings demonstrate sex‐related differences in intestinal community composition and diversity as a result of the administration of both GcL and SsoPox, with SsoPox having a slightly greater effect overall. These results may suggest a competitive dynamic between certain QQ molecules, their AHL substrates, the microbiota, and host hormone regulation. Further research is necessary to determine if these observations are due to differences in endocrine signaling between male and female mice.

The limitations of this study include small sample sizes, testing only in a single genotype, limited dose range, and no observed phenotypic effect. Although we were able to apply the SpPDCC package for mice treated with 1 mg/mL doses, our sample size testing 2 mg/mL doses was too small to apply this tool. Future studies should employ larger sample sizes to further assess the biological applicability of the SpPDCC package for covariance network analysis.^61^ Additionally, our study employed the use of two QQ lactonases at dosages of 1‐ and 2 mg/mL. Given the amplification of effects on community composition and diversity with the 2 mg/mL dose, our results suggest that higher doses of QQ lactonases may yield a greater degree of change in composition and diversity in the intestinal microbiota. However, due to the small sample size, additional testing is needed to determine definitive dose responses, and future studies will be needed to investigate the optimal clinical dose for potential therapeutic applications of QQ lactonases. Additionally, testing in multiple mouse genotypes will be necessary to establish effective dose ranges and safety profiles across different backgrounds. Finally, treatment with QQ lactonases did not produce a significant phenotypic effect, measured as changes in weight and food intake, in a murine model of diet‐induced obesity over a 6‐week period. Further research should monitor caloric intake and energy expenditure to better assess the effects of QQ lactonase treatment on host phenotype. Moreover, changes in metabolomic and transcriptomic profiles should be investigated to address changes in microbial function that could be related to maintenance of intestinal homeostasis.^62^

Overall, the two tested QQ lactonases exhibit separate, sex‐dependent and dose‐dependent effects on the intestinal microbiota, demonstrating proof of concept that QQ is a targetable strategy for microbial control. Further characterization and dosage optimization of QQ enzymes may reveal therapeutic applications for a variety of chronic microbial‐associated diseases.

## AUTHOR CONTRIBUTIONS

**Aneesh Syal:** Data curation; formal analysis; investigation; software; writing – original draft. **Maria Martell:** Data curation; formal analysis; investigation; software; writing – original draft. **Rakesh Sikdar:** Methodology; resources; writing – review and editing. **Matthew Dietz:** Data curation; investigation; software; writing – review and editing. **Zachary Ziegert:** Investigation; writing – review and editing. **Cyrus Jahansouz:** Investigation; writing – review and editing. **Mikael H. Elias:** Conceptualization; funding acquisition; project administration; resources; supervision; writing – review and editing. **Christopher Staley:** Conceptualization; funding acquisition; project administration; supervision; writing – review and editing.

## FUNDING INFORMATION

Support from the Biotechnology Institute and the MnDrive initiative (to MHE) is acknowledged.

## CONFLICT OF INTEREST STATEMENT

Mikael H. Elias is a co‐founder, a former CEO, and an equity holder of Gene&GreenTK, a company that holds the license to WO2014167140 A1, FR 3068989 A1, and FR 19/02834. He has filed the patents EP3941206 and WO2020185861A1. His interests with Gene&GreenTK have been reviewed and managed by the University of Minnesota in accordance with its conflict‐of‐Interest policies. The remaining authors declare that the research was conducted in the absence of any commercial or financial relationships that could be construed as a potential conflict of interest.

## ETHICAL STATEMENT

Experimental procedures were approved by the University of Minnesota Institutional Animal Care and Use Committee (IACUC) and performed following the Office of Laboratory Animal Welfare guidelines (ethical approval number: 2206‐40179A).

## Data Availability

Raw sequencing data were deposited in the Sequence Read Archive under BioProject accession number SRP415730.
